# Anogenital Erosive Lichen Sclerosus in a Male With Multiple Autoimmune Conditions

**DOI:** 10.7759/cureus.64908

**Published:** 2024-07-19

**Authors:** Mohammad I Fardos, Jacqueline Nikakis, Sophia Anagnostis, Summer Moon

**Affiliations:** 1 Dermatology, HCA Florida Largo Hospital, Largo, USA; 2 General Medicine, Peconic Bay Medical Center/Donald and Barbara Zucker School of Medicine at Hofstra/Northwell, Riverhead, USA

**Keywords:** hypertrophic lichen planus, anogenital, clinical dermatology, auto immune, lichen sclerosus

## Abstract

Lichen sclerosus (LS) is a chronic inflammatory disorder primarily affecting the anogenital region, with a higher prevalence in females and often linked to autoimmunity. This association is not clearly elucidated in males, with LS commonly presenting in uncircumcised males. The most affected areas include the glans penis, prepuce, and coronal sulcus. In this report, we present an 11-year case of treatment-resistant LS in a male patient with an extensive history of autoimmune disorders, manifesting in the intergluteal cleft as a hypertrophic plaque, a rare location. The patient had a complex autoimmune history, including porphyria cutanea tarda, discoid lupus, and Sjogren's syndrome. Histopathological analysis confirmed a diagnosis of erosive LS. Despite numerous treatments, including intralesional corticosteroids and various topicals, the lesion persisted. This case highlights the challenges in managing LS, particularly in uncommon sites and in patients with extensive autoimmune backgrounds. Treatment goals for LS focus on symptom relief, cosmetic improvement, and disease prevention. Although topical corticosteroids are commonly used, systemic options like hydroxychloroquine may be beneficial in resistant cases, although clear guidelines are lacking. Our case underscores the importance of a multidisciplinary approach in addressing LS and its associated autoimmune conditions.

## Introduction

Lichen sclerosus (LS) is a chronic inflammatory condition that typically affects the anogenital region [1]. LS predominantly affects females at a ratio of 5:1 and is associated with autoimmune diseases such as autoimmune thyroid disease, vitiligo, and psoriasis [2]. This association is not clearly elucidated in males, with LS commonly presenting in uncircumcised males. The most commonly affected areas in males include the glans penis, prepuce, and coronal sulcus [2]. Here, we present a case of treatment-refractory LS in a male patient with an extensive autoimmune history, occurring in the intergluteal cleft, a unique location.

## Case presentation

A 56-year-old African American man presented in the summer of 2023 with a chief complaint of a rash affecting his intergluteal cleft, which had been present for the past eleven years. His past medical history was significant for porphyria cutanea tarda (PCT) and discoid lupus erythematosus (DLE), both diagnosed in 2012. The patient's DLE was treated with topical corticosteroids, and his PCT was managed with phlebotomy.

Historically, the patient was initially evaluated in the spring of 2012 for an intensely pruritic rash of his intergluteal cleft. A shave biopsy at that time revealed lichen simplex chronicus. He was treated with 5 mg of intralesional Kenalog (ILK), high-potency topical corticosteroids, topical calcineurin inhibitors, and zinc barrier cream, with minimal improvement. Due to persistent symptoms, the patient had a repeat shave biopsy in 2015, which revealed lichenoid interface dermatitis, compatible with lichen planus. Further autoimmune work-up in 2016 revealed a positive antinuclear antibody (ANA) titer, positive rheumatoid factor, and elevated serum porphyrin levels. The patient was further evaluated by rheumatology, and systemic lupus and rheumatoid arthritis were ruled out. The patient was lost to follow-up between 2016 and 2023 before presenting again in the summer of 2023. Systemic treatment with hydroxychloroquine was considered at that time but ultimately not started due to patient preferences.

**Figure 1:**
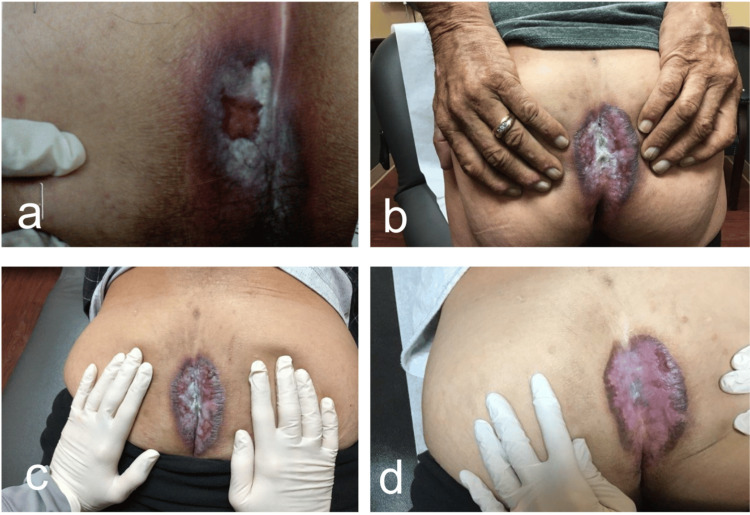
Progression of Perianal Plaque (a) Spring of 2012; (b) summer of 2023 showing a well-demarcated plaque; (c) fall of 2023; (d) winter of 2024: hypertrophic and lichenified plaque on the left gluteal fold, approximately 7 to 8 cm in diameter.

**Figure 2:**
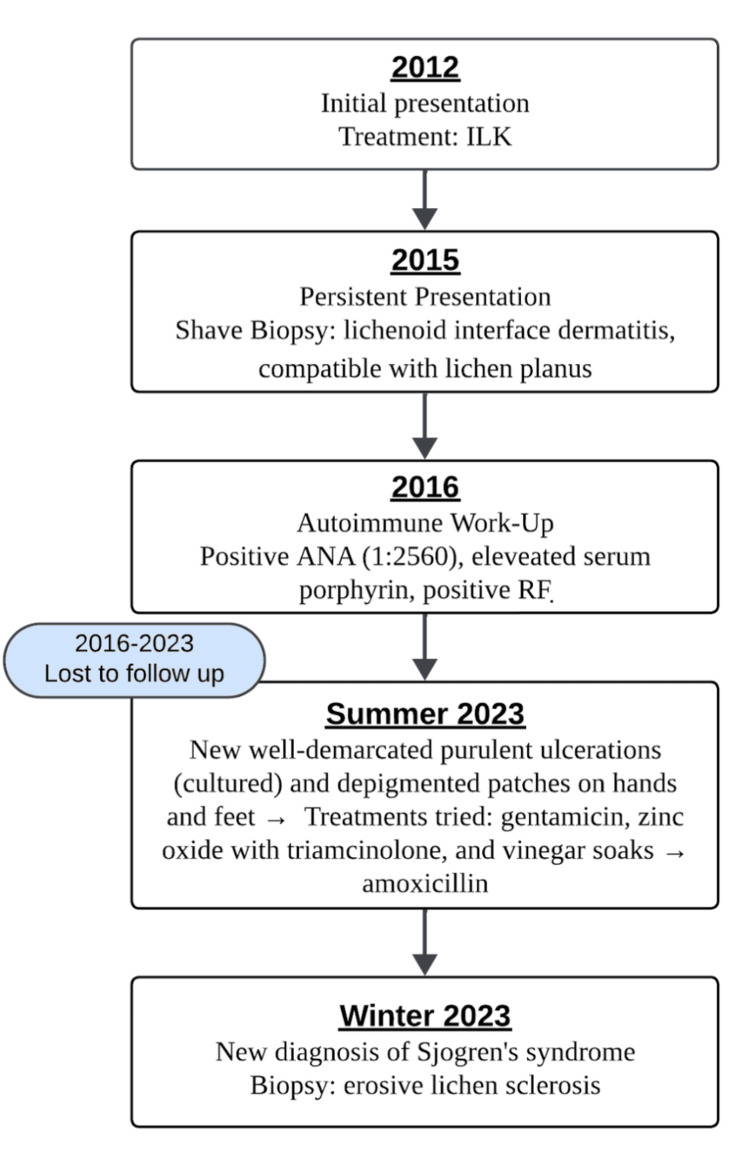
Timeline detailing disease progression ILK: intralesional Kenalog, ANA: antinuclear antibody, RF: rheumatoid factor.

In the summer of 2023, physical examination revealed a hypertrophic and lichenified plaque on the left gluteal fold, approximately 7 to 8 cm in diameter (Figures 1 and 2). The patient reported associated intense pruritus. A shave biopsy was performed, which revealed parakeratosis with crust overlying the epidermis, along with acanthosis featuring hypergranulosis and necrotic keratinocytes at the base. Furthermore, a dense dermal infiltrate predominantly composed of lymphocytes was noted. The Fite, PAS (periodic acid-Schiff), and GMS (Gomori methenamine-silver) stains were negative. Collectively, these findings were suggestive of erosive LS. The patient had a repeated rheumatology work-up, which revealed a positive ANA titer with a speckled pattern, and was diagnosed with Sjogren’s syndrome. The patient was started on ILK 10 mg with improvement in pruritus in the winter of 2023. However, due to the persistent hypertrophic plaque despite multiple treatments with ILK and various topical therapies, the patient is now being considered for systemic hydroxychloroquine treatment. The patient has not been treated with systemic therapies for any of his autoimmune diseases, including DLE, PCT, and Sjogren's syndrome.

## Discussion

Treatment goals in LS aim to alleviate symptoms, improve appearance, and prevent disease progression and complications. Topical therapies play a crucial role in managing anogenital LS. High-potency topical corticosteroids are first-line medications for LS due to their robust anti-inflammatory effects, effectively mitigating pruritus, pain, and inflammation. Alternatively, calcineurin inhibitors, specifically tacrolimus and pimecrolimus, have demonstrated high efficacy in treating LS in instances where corticosteroids are contraindicated [3]. In addition to topical therapies, localized steroid injections may be utilized to manage thicker lichenified plaques.

In cases refractory to topical treatment, systemic modalities may be considered; however, there are no established guidelines. A review of 71 studies encompassing 392 LS patients, including those with anogenital (254), extragenital (36), and both types (73) of LS, found that systemic therapies such as oral retinoids, methotrexate, hydroxychloroquine, and systemic steroids resulted in clinical or symptomatic improvement in a significant proportion of patients [3]. Notably, 76% of patients with anogenital involvement and 81% of patients with both types of LS experienced improvements following systemic treatment [4]. Despite these findings, established guidelines for systemic LS treatment remain lacking.

Hydroxychloroquine may be useful in the treatment of our patient's treatment-resistant anogenital LS as well as other concurrent autoimmune diseases, including DLE, PCT, and Sjogren's syndrome.

## Conclusions

This case underscores the rarity of LS manifesting in a male patient with an extensive autoimmune history and occurring in a unique location, the intergluteal cleft. It additionally highlights the challenges posed by treatment-resistant LS. Despite various treatment attempts, including topical corticosteroids and intralesional injections, the plaque persisted. While topical therapies remain the cornerstone of treatment, systemic options such as hydroxychloroquine may offer promise in refractory cases, although clear guidelines are currently lacking. Further investigation is warranted to elucidate optimal management strategies for erosive LS, particularly in patients with complex autoimmune backgrounds like the one presented here.
